# Egg on a string sign

**DOI:** 10.11604/pamj.2018.31.79.17024

**Published:** 2018-10-03

**Authors:** Ipek Guney Varal, Pelin Dogan

**Affiliations:** 1 Department of Pediatrics, Division of Neonatology, University of Health Sciences, Bursa Yüksek Ihtisas Teaching Hospital, Bursa, Turkey

**Keywords:** Congenital heart disease, chest X-ray, newborn

## Image in medicine

A 2850g male infant was born at 36 weeks gestation to a 26-year-old gravida 1 para 1 mother who did not receive routine prenatal care. Upon delivery he was hospitalized to the neonatal intensive care unit for severe respiratory distress. On physical examination, the infant had marked central cyanosis and a soft systolic murmur. Chest X-ray showed “egg on a string” sign raising suspicion for transposition of the great arteries, which was confirmed by urgent echocardiography and cardiac catheterization. Transposition of the great arteries is the most common cyanotic congenital heart lesion that presents in neonates. The hallmark of the condition is ventriculoarterial discordance and the classic “egg on a string” appearance on chest roentgenograms is found in one third of patients. A continuous infusion of prostaglandin E1 is the mainstay of emergent treatment followed by surgical arterial switch procedure.

**Figure 1:**
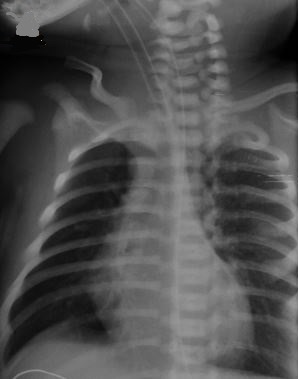
Egg on a string sign in chest X-ray

